# Construction of Condition-Specific Gene Regulatory Network Using Kernel Canonical Correlation Analysis

**DOI:** 10.3389/fgene.2021.652623

**Published:** 2021-05-20

**Authors:** Dabin Jeong, Sangsoo Lim, Sangseon Lee, Minsik Oh, Changyun Cho, Hyeju Seong, Woosuk Jung, Sun Kim

**Affiliations:** ^1^Interdisciplinary Program in Bioinformatics, Seoul National University, Seoul, South Korea; ^2^Bioinformatics Institute, Seoul National University, Seoul, South Korea; ^3^BK21 FOUR Intelligence Computing, Seoul National University, Seoul, South Korea; ^4^Department of Computer Science and Engineering, Seoul National University, Seoul, South Korea; ^5^Department of Crop Science, Konkuk University, Seoul, South Korea; ^6^Department of Computer Science and Engineering, Institute of Engineering Research, Seoul National University, Seoul, South Korea

**Keywords:** kernel canonical correlation analysis, gene regulatory network, network dynamics, transcription factor, TF cooperation, condition specific network

## Abstract

Gene expression profile or transcriptome can represent cellular states, thus understanding gene regulation mechanisms can help understand how cells respond to external stress. Interaction between transcription factor (TF) and target gene (TG) is one of the representative regulatory mechanisms in cells. In this paper, we present a novel computational method to construct condition-specific transcriptional networks from transcriptome data. Regulatory interaction between TFs and TGs is very complex, specifically multiple-to-multiple relations. Experimental data from TF Chromatin Immunoprecipitation sequencing is useful but produces one-to-multiple relations between TF and TGs. On the other hand, co-expression networks of genes can be useful for constructing condition transcriptional networks, but there are many false positive relations in co-expression networks. In this paper, we propose a novel method to construct a condition-specific and combinatorial transcriptional network, applying kernel canonical correlation analysis (kernel CCA) to identify multiple-to-multiple TF–TG relations in certain biological condition. Kernel CCA is a well-established statistical method for computing the correlation of a group of features vs. another group of features. We, therefore, employed kernel CCA to embed TFs and TGs into a new space where the correlation of TFs and TGs are reflected. To demonstrate the usefulness of our network construction method, we used the blood transcriptome data for the investigation on the response to high fat diet in a human and an arabidopsis data set for the investigation on the response to cold/heat stress. Our method detected not only important regulatory interactions reported in previous studies but also novel TF–TG relations where a module of TF is regulating a module of TGs upon specific stress.

## 1. Introduction

In a living cell, rewiring of interactions among proteins, genes, and RNA molecules orchestrates how cells respond to external stimuli. One of the most fundamental regulatory relationships arise from transcription factors (TFs) that bound to the promoter of target genes (TGs) resulting in changing transcriptional dynamics. Since TF–TG interactions can be represented as a network, dynamics of gene regulatory mechanisms upon stimuli can be modeled and analyzed as gene regulatory network (GRN). High-throughput experimental techniques, such as Chromatin Immunoprecipitation sequencing (ChIP-seq), have been widely utilized to construct GRNs detecting one-to-multiple relationships of TF and TGs (i.e., relations of a TF and the promoters of TGs where the TF binds to). Such experimental techniques are powerful but provide only partial snapshot of condition-specific GRN. TF ChIP-seq can measure only one TF at a time and it is not practical to perform ChIP-seq experiments for all TFs under various conditions. More importantly, multiple TFs work together to regulate multiple TGs in a condition-specific way, thus data from TF ChIP-seq needs to be combined for constructing networks of multiple TFs and multiple TGs simultaneously. Thus, it is necessary to develop computational methods for elucidating multiple-to-multiple relations of TFs and TGs in a specific condition. There have been several studies to identify multiple-to-multiple interactions. A study by Jolma et al. (2015) tried to identify TF–TG regulations using a tailored experimental technique in a multiple-to-multiple fashion. Their work is still limited in identifying only 315 TF–TF interactions from ~2,000 putative TFs.

There have been growing attention in *in silico* reverse engineering methods that infer GRNs from gene expression data. Correlation-based network inference methods—the most straightforward approach—detect regulatory relations if two genes are linearly correlated (Eisen et al., 1998). However, the correlation-based methods are prone to produce many false-positive relations, i.e., the relations predicted by computational methods but not detected in experimental validations, because the methods consider solely a linearly correlated expression pattern between a pair of genes. For example, if two genes *B* and *C* are regulated by a common gene *A*, expression patterns of *B* and *C* are correlated thus detected as regulatory relations even though there are no direct regulatory relationships between *B* and *C*. A number of computational methods with different strategies have been developed over two decades. Methods based on mutual-information (MI) is a generalization of correlation-based model that can detect non-linear dependencies, taking into account the effect of third-party genes in addition to two correlating genes. ARACNe (Margolin et al., 2006) and ARACNe-AP, one of the most popular reverse engineering methods, use the data-processing inequality to prune the indirect regulations if a pair of genes interact only through a third gene in every possible gene triplets. Likewise, the three-way mutual information (MI3) and conditional mutual information (CMI)-based models consider the effect of co-regulators in order to remove false-positive interactions (Luo et al., 2008; Zhang et al., 2012). Besides, regression-based methods considers multiple-to-one relations of TFs and a TG as a feature selection problem, where the expression of TGs is predicted from the expression of all other TF genes (Xiong and Zhou, 2012; Hill et al., 2016). GENIE3, one of the most best-performing methods, utilized an ensemble of regression trees to select putative TFs for each TG. Although MI-based approaches showed lower false-positive rate than correlation-based methods, they do not consider the biological nature of TFs—combinatorial and cooperative nature of TFs—when regulating TGs are disregarded.

Then, how TFs work in order to coordinate certain biological functions? First, TFs regulate a biological function through interacting with protein complexes rather than simply elevating mRNA concentration (Sutherland and Bickmore, 2009; Rieder et al., 2012; Duren et al., 2019). Therefore, to detect important TFs that are related to a certain biological function, TF interaction network should be utilized rather than simply detecting TFs with the highest mRNA concentration. Second, combinatorial interaction of TFs regulates TGs to control certain biological functions. That is, given alternative stimuli, different combinations of TFs may regulate expression of different sets of TGs to certain cellular response involving multiple-to-multiple relations of TFs and TGs. Several studies have suggested an atlas of combinatorial TF module interactions (Ravasi et al., 2010; Wise and Bar-Joseph, 2015; Guo and Gifford, 2017) and inferred their associated regulators using probabilistic graph models (Segal et al., 2003).

In this paper, we present a new computational method that reconstructs GRN from gene expression data incorporating the aforementioned biological nature of TFs. We detected cooperating TFs that coordinate common biological functions utilizing public protein–protein interaction (PPI) network. For detection of combinatorial relations of TFs and TGs specific to the dataset, i.e., condition-specific combinatorial relations, we utilized kernel canonical correlation analysis (kernel CCA). Kernel CCA is a well-established statistical method for learning coefficients of two groups of features that maximize the correlation of a group of features vs. another group of features (Kuss and Graepel, 2003; Akaho, 2006; Rhee et al., 2009; Ashad Alam and Fukumizu, 2015; Richfield et al., 2016; Tang et al., 2019). A high value of coefficients or weights of features implies that the features from different groups are relevant. For example, applying kernel CCA in motif data and gene expression data, features (e.g., motif) with high weights are deduced as relevant motifs in regulating gene expression (Rhee et al., 2009). Therefore, conducting kernel CCA on gene expression data consisting of groups of features—one feature set composed of TFs and another feature set composed of TGs—can detect TF–TG regulatory relations. Specifically, we employed kernel CCA to embed TFs and TGs into a new space where the correlation of TFs and TGs are reflected to detect context-specific, i.e., response to external stimulus, TF–TG relations. This enables the construction of GRN that models responses to stimuli shows dynamics of GRN over time, applying our method in time-series data. Since we utilized PPI network to detect co-working TFs, we can modularize a GRN into sub-networks of manageable size, which resulted in the improved interpretability of GRN.

## 2. Method

**Figure 1:**
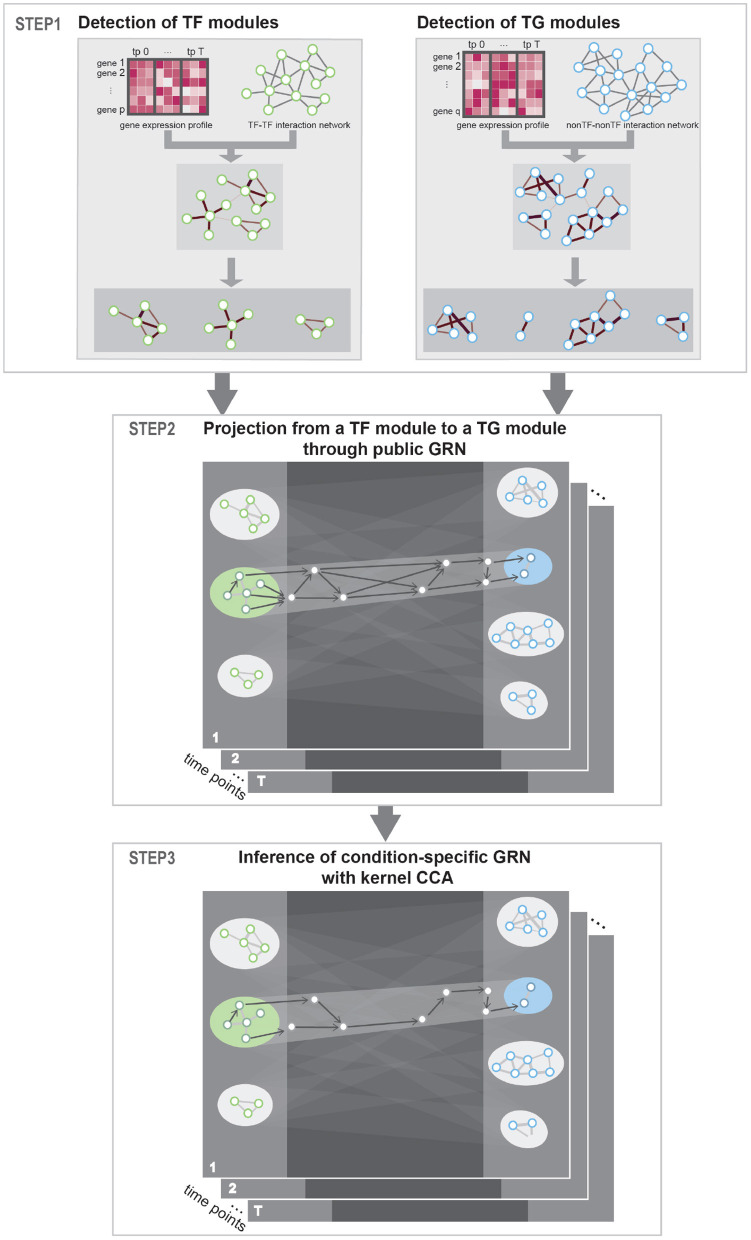
Workflow. STEP 1: To detect interacting transcription factor (TF) and target gene (TG) modules, respectively, prior protein–protein interaction (PPI) network was instantiated with gene expression data and community detection algorithm was used to detect condition-specific TF and TG modules. STEP 2: To get putative TF–TG relations, we conducted projection from a TF module to a TG module through public gene regulatory network (GRN). This process is conducted for every possible TF–TG module pair. STEP 3: Utilizing kernel canonical correlation analysis (CCA), we constructed condition-specific GRN that detects multiple-to-multiple regulatory relationships between TFs and TGs.

The method proposed in this paper aimed at constructing condition-specific GRNs considering the cooperative and combinatorial nature of TFs. To detect cooperative TFs that share common biological process, we utilized PPI network as a prior knowledge. Then, to detect combinatorial multiple-to-multiple regulatory relations between TFs and TGs, we utilized kernel CCA in inferring regulatory interactions. Our approach uses gene expression profile data in multiple conditions (e.g., time points) as input and produces a network of gene–gene regulatory relations. Public PPI network and GRN network were utilized as a prior knowledge to guide the detection of correct TF–TG relations. Specifically, our approach consists of three steps—**Step 1:** Identification of TFs and TGs modules. **Step 2:** Construction of regulator relationships among the TF/TG modules. **Step 3:** Inference of condition-specific GRN—as described in Figure 1.

### 2.1. STEP 1: Identification of TF and TG Modules

Since TFs work as a protein complex or as a group to direct common biological functions (Sutherland and Bickmore, 2009; Rieder et al., 2012; Duren et al., 2019), we aimed at identifying a group of TFs that work together and TGs that are regulated by the TFs. Genes were classified as TFs referring to the public TF catalogs (Jin et al., 2016; Lambert et al., 2018), otherwise as TGs. We used PPI network—STRING (v10.5) (Szklarczyk et al., 2016) and BioGrid (v.3.5.179) (Stark et al., 2006) database—as putative interactions of genes. STRING database compiled interaction based on experimental data or from the literature. Some interactions in STRING are made by using computational prediction methods, which may contain many false-positive interactions. On the other hand, BioGrid primarily compiled experimentally validated interactions. Thus, interactions in BioGrid may be more reliable but inference using BioGrid may suffer a high level of false-negatives. We concatenated both of the databases to complement each other's limitations. Then, we filtered the network with TFs to build TF–TF interaction network and with TGs to build non-TF–non-TF interaction network (i.e., TG–TG interaction network). In our study, these two networks are used as template networks of co-working or interacting genes. To detect condition-specific network of TFs and TGs for a given context, we instantiated the TF–TF interaction network with expression data of TFs and the TG–TG interaction network with expression data of non-TFs (Ahn et al., 2017). In particular, among gene–gene interactions in template networks, interactions whose Pearson's correlation coefficient between expression vector of corresponding genes below 0.5 are discarded. Using condition-specific networks, respectively, we detected clusters of TFs ad TGs with a multi-level community detection algorithm to detect condition-specific TFs and TG modules. We utilized multilevel.community function in R igraph package that implemented the Louvain algorithm for community detection (Csardi and Nepusz, 2006).

### 2.2. STEP 2: Construction of Preliminary GRN Between TF and TG Modules

A very large search space of TF–TG relationships is one of the challenges in reverse engineering of GRN. Given *n* genes, *n*^2^ combinations of interactions should be considered. In particular, it is not computationally feasible to perform kernel CCA analysis on a very large network. Even if it is feasible, no computational methods can produce correct results when there are many unknown factors, true relations in this case. To reduce search space, we used publicly reported gene regulatory relationships as a guide to navigate TF–TG relationships. Specifically, we merged public GRNs: TRRUST (Han et al., 2018) and HTRIdb (Bovolenta et al., 2012), computationally predicted TF-DNA-binding sites data (Ernst et al., 2010) for Human dataset; PlantRegMap (Tian et al., 2020) and ATRM (Jin et al., 2015) for Arabidopsis dataset. Then, we pruned the network with genes with signature genes—for example, differentially expressed genes (DEGs) or genes with high variance across samples—to navigate the GRN in condition-specific perspectives. A subgraph of GRN that contained signature genes and their first nearest neighbors in public GRNs is utilized as condition-specific gene regulation candidates. For every combination of TF modules and TG modules, projection from a TF cluster to a TG cluster through all shortest paths in the GRN yields a sub-network of GRN and we utilized these edges from the sub-network as a TF–TG relationship candidate.

### 2.3. STEP 3: Inference of Condition-Specific GRN With Kernel CCA

For each of preliminary sub-network of GRN determined in section 2.2, our goal is to construct condition-specific sub-networks considering multiple-to-multiple relationships of TFs and TGs. Specifically, we utilized kernel CCA to embed TFs and TGs in canonical dimensions. Then, we measured cosine similarity between TFs and TGs in the embedding space to discover TF–TG pairs that contribute to the correlation between the groups of TFs and TGs. Since TFs can also regulate expression of other TFs, which in turn generate TF cascading network, we iteratively conducted kernel CCA embedding and TF–TG relation detection for every possible relationship in each GRN sub-network.

#### 2.3.1. Kernel Canonical Correlation Analysis

A common biological phenomenon shared by groups of genes tends to yield a high correlation detected between expression vectors of the genes (Yamanishi et al., 2003; Rhee et al., 2009). CCA is a method to detect shared correlation across variables from heterogeneous datasets and yield canonical vectors, which are weight coefficients for linear combination of variables in each dataset. These canonical vectors represent how much contribution or weights each variable has in correlation. Kernel CCA is a generalized version of CCA that can detect non-linear relationships between variables. Therefore, we utilized regularized kernel CCA (Bilenko and Gallant, 2016) to retrieve new embedding of TFs and TGs that reflects contribution of genes in correlation between expression level of TFs and TGs. Highly scored TFs and TGs in canonical vectors are considered as genes that contribute to correlation of shared biological phenomenon between TFs and TGs.

Let $\text{X}=\left({\text{x}}_{1},{\text{x}}_{2},\ldots ,{\text{x}}_{n}\right)\in {\mathbb{R}}^{n\times p}$ and $\text{Y}=\left({\text{y}}_{1},{\text{y}}_{2},\ldots ,{\text{y}}_{n}\right)\in {\mathbb{R}}^{n\times q}$ be the gene expression matrices of TFs and TGs with *n* samples and *p* genes and with *n* samples and *q* genes, respectively. The original gene expression profiles are mapped to high-dimensional feature space, reproducing kernel Hilbert space (RKHS), through feature maps $\phi_{x}:\text{x}\in {\mathbb{R}}^{p}\mapsto H_{x}$ and $\phi_{y}:\text{y}\in {\mathbb{R}}^{q}\mapsto H_{y}$. Feature vector ϕ_*x*_(**x**) is the projection of a data point **x** ∈ **X** and likewise ϕ_*x*_(**x**) is the projection of a data point **y** ∈ **Y**. We represent the datasets projected in feature space as Φ_*x*_ = ϕ_*x*_(**x**_1_), ϕ_*x*_(**x**_2_), ⋯ , ϕ_*x*_(**x**_*n*_) and Φ_*y*_ = ϕ_*y*_(**y**_1_), ϕ_*y*_(**y**_2_), ⋯ , ϕ_*y*_(**y**_*n*_), respectively. Applying kernel trick, the similarities of feature vectors can be defined as a positive definite kernel $k_{x}\left({\text{x}}_{i},{\text{x}}_{j}\right)={\langle \phi_{x}\left({\text{x}}_{i}\right),\phi_{x}\left({\text{x}}_{j}\right)\rangle }_{H_{x}}$ and $k_{y}\left({\text{y}}_{i},{\text{y}}_{j}\right)={\langle \phi_{y}\left({\text{y}}_{i}\right),\phi_{y}\left({\text{y}}_{j}\right)\rangle }_{H_{y}}$, where *i, j* = 1, 2, …, *n*. Specifically, we applied Gaussian RBF kernel (Equation 1)

(1) $$\begin{array}{l} k_{x}(x_{i},x_{j})=\exp\left[-\frac{\|x_{i}-x_{j}{\|}^{2}}{2\sigma^{2}}\right]\\ k_{y}(y_{i},y_{j})=\exp\left[-\frac{\|y_{i}-y_{j}{\|}^{2}}{2\sigma^{2}}\right] \end{array}$$

We define kernel projection of data or kernel Gram matrices as ${\text{K}}_{x}={\left(k_{x}\left({\text{x}}_{i},{\text{x}}_{j}\right)\right)}_{i,j=1}^{n}=\Phi_{x}^{T}\Phi_{x}$ and ${\text{K}}_{y}={\left(k_{y}\left({\text{y}}_{i},{\text{y}}_{j}\right)\right)}_{i,j=1}^{n}=\Phi_{y}^{T}\Phi_{y}$.

The aim of kernel CCA is to find projection vectors *f*_*x*_ and *f*_*y*_ that maximize the correlation of canonical components $\text{u}={\langle f_{x},\phi_{x}\left(\text{x}\right)\rangle }_{H_{x}}$ and $\text{v}={\langle f_{y},\phi_{y}\left(\text{y}\right)\rangle }_{H_{y}}$. Since canonical vectors *f*_*x*_ and *f*_*y*_ lie in space spanned by the feature space mapped objects, we can represent canonical vectors as linear combinations of Φ_*x*_ and Φ_*y*_, where $f_{x}=\Phi_{x}^{T}\alpha$ and $f_{y}=\Phi_{y}^{T}\beta$. Therefore, canonical components *u* and *v* are represented with kernel matrix, $u=\Phi_{x}^{T}\Phi_{x}\alpha ={\text{K}}_{x}\alpha$ and $v=\Phi_{y}^{T}\Phi_{y}\beta ={\text{K}}_{y}\beta$. The objective function of the kernel CCA is restated with kernel projections as follows:

(2) $$\underset{{}_{\alpha ,\beta }}{\text{argmax}}\text{ corr}(u,v)=\underset{{}_{\alpha ,\beta }}{\text{argmax}}\;\alpha \;K_{x}\;K_{y}\;\beta$$

where α ∈ ℝ^*n*^,β ∈ ℝ^*n*^ are expansion coefficients. The problem can be reformulated as a generalized eigenvalue problem with regularization as follows:

(3) $$\left(\begin{array}{cc} 0 & K_{x}K_{y}\\ K_{y}K_{x} & 0 \end{array}\right)\left(\begin{array}{c} \alpha \\ \beta \end{array}\right)=\rho^{2}\left(\begin{array}{cc} K_{x}^{2}+\lambda I & 0\\ 0 & K_{y}^{2}+\lambda I \end{array}\right)$$

where **I** denotes the identity matrix, λ is regularization parameter, and ρ = max〈*u, v*〉/(∥*u*∥∥*v*∥). Once we obtain solutions for the above equations that represent the amount of contribution of each sample, we multiplied the transpose of gene expression matrices **X**^*T*^ ∈ ℝ^*p*×*n*^ and **Y**^*T*^ ∈ ℝ^*q*×*n*^ with canonical weight vectors α ∈ ℝ^*n*^ and β ∈ ℝ^*n*^ to get the TF and TG embeddings, $w_{x}\in {\mathbb{R}}^{p}$ and $w_{y}\in {\mathbb{R}}^{q}$ that represents the amount of contribution of each gene (Equation 4).

(4) $$\begin{array}{l} w_{x}=X^{T}\;\alpha \\ w_{y}=Y^{T}\;\beta \end{array}$$

We can now compute *k* canonical components orthogonal to each other, so that we can get TF and TG embeddings matrix ${\text{W}}_{x}\in {\mathbb{R}}^{p\times k}$ and $W_{y}\in {\mathbb{R}}^{q\times k}$ where each row in matrix stands for new embeddings of TGs and TGs in *k* canonical dimensions.

#### 2.3.2. Detection of Multiple-To-Multiple Relations of TFs and TGs

Using kernel CCA, genes that greatly contribute to the correlations of TFs and TGs gain greater weights in canonical embeddings and TF–TG pair that both TF and TG show high weights should be remarked as valid pair. Inspired by Seo and Kim (2013), we weighted every *k* dimension with the corresponding eigenvalue so that the eigenvalue-weighted embeddings is dominated by the leading eigenvectors. For every possible TF–TG pair retrieved from public GRN, we next computed dot-product similarity of TF and TG embeddings to define an edge weight of the pair. We then filtered out edges that have weights below 0.5. This process is iteratively performed until there are no TFs left in candidate TG lists.

## 3. Data and Performance Evaluation Scheme

### 3.1. Data

We analyzed public time-series gene expression data from NCBI GEO datasets (GSE127530, GSE5621, and GSE5628).

- GSE127530 is an RNA-seq data that measure human blood transcriptome after high-fat meal (HFM) measured in three time points (Fast, +3, and +6 h after stimulus) with 15 samples for each time point, where each time point denoted as tp0, tp1, and tp3. Raw counts are normalized in terms of gene length with TPM (transcripts per million). For our method, we applied MinMaxscaler in Python sklearn library in order not to make correlations dominated by highly expressed genes
- GSE5621 is an microarray data that measures transcriptome from shoots in *Arabidopsis thaliana* in response to cold stress at seven time points (0, +0.5, +1, +3, +6, +12, and +24 h) with two replicates for each time point, where each time point denoted as tp0, tp1, tp2, tp3, tp4, tp5, and tp6. GSE5628 is an microarray data responsive to heat stress, which consists of heat-shocked samples at 38 Centigrade and recovered samples after heat-shock treatment prolongs to 21 h at 25 Centigrade measured at five time points (0, +0.25, +0.5, +1, and +3 h) with two replicates for each time point, where each time point denoted as tp0, tp1, tp2, tp3, and tp4. We applied MinMaxscaler in Python sklearn library for normalization.

### 3.2. Evaluation 1: Performance Comparison With Existing Methods

We compared our method with the existing methods: ARACNe-AP (Lachmann et al., 2016) and GENIE3 (Irrthum et al., 2010). ARACNe-AP is a representative reverse engineering method based on information theoretic approach for GRN construction while GINIE3 uses a regression tree method. We then compared how much condition-specific signature each method can capture, utilizing GSE127530 dataset. ARACNe-AP does not yield valid edges from the datasets, thus GRN constructed with GSE5621 and GSE5628 datasets were excluded.

- **Construction of Ground Truth GRN**: To assess the network inference performance, we constructed condition-specific GRN as a ground truth gene set. A comprehensive biomedical entity search tool, BEST (Lee et al., 2016), was utilized to retrieve condition-specific gene sets using four keywords from literature search related to HFM: “lipid metabolism (Ming et al., 2009),” “obesity (Golay and Bobbioni, 1997),” “diabetes (Salmeron et al., 2001; Marshall and Bessesen, 2002),” and “innate immunity (McLaughlin et al., 2017; Childs et al., 2019).” Among the four keywords, “innate immunity” is the term reported as a related biological term in the paper that reported the GSE127530 dataset (Lemay et al., 2019). The literature search identified 1,131 HFM-associated genes. These genes were mapped according to the public GRN described in section 2.2. As a result, we constructed a ground truth network of 738 nodes and 1,991 edges that connect 2 HFM-associated genes.
- **Metrics for Performance Measurements**: Given the nodes and edges of an inferred GRN by our method, we measured the overlap of the nodes and edges between the inferred GRN and the ground truth GRN.
  − specificity = TN/(TN+FP)
  − precision = TP/(TP+FP)
  − recall = TP/(TP+FN)
  For the node-level comparison, we measured specificity and recall. True-positive (TP) are a set of genes that are both in the ground truth network and reported by our method. False-positive (FP) is a set of genes that are reported by our method but do not exist in the ground truth network. True-negative (TN) is a set of genes that are not in the ground truth network and not reported by our method. False negative (FN) is a set of genes that are not reported by our method but exist in the ground truth network. For the edge-level comparison, we measured precision and recall. TP are a set of edges that are both in the ground truth network and reported by our method. FP is a set of edges that are reported by our method but do not exist in the ground truth network. FN is a set of edges that are not reported by our method but exist in the ground truth network.

### 3.3. Evaluation 2: Investigation of TF Cooperation

A sub-network constructed by our method contains multiple TFs that cooperate with each other for regulating TGs in the sub-network. One way to evaluate the power of TF cooperation is to compare metrics from all TFs in the sub-network vs. metrics from a set of individual TFs in the sub-network. That is, we constructed sub-networks using individual TFs in TF modules without considering the cooperativeness of TFs. The original sub-network (denoted as *G*_*all*_) that was constructed using all cooperating TFs in TF modules was compared to the sub-networks (each sub-network denoted as *G*_*i*_) that was constructed using individual TFs in TF modules. We used two metrics for the evaluation of TF cooperation: the biological significance and the cooperative potential.

#### 3.3.1. Biological Significance

Biological significance (*B*_*p*_) of TF cooperation in terms of each pathway was calculated using Equation (5). Pathway enrichment with nodes in *G*_*all*_ and all $G_{i}^{\prime }s$ were was calculated using Enrichr (FDR < 0.05) (Chen et al., 2013) in gseapy library. For each pathway *p*, the *p*-value obtained from *G*_*all*_ is denoted as $p_{a}^{p}$ and the *p*-value obtained from *G*_*i*_ is denoted as $p_{i}^{p}$. Since multiple *G*_*i*_s are constructed, aggregating pathway *p*-values from *G*_*i*_ was performed by Fisher's combined probability test (Fisher, 1992). Specifically, a set of *p*-values from *k* independent tests to calculate a test statistic $\chi_{F}^{2}=-2\sum_{i=1}^{k}\left[\ln[p_{i}^{p}\right]$ that follows χ^2^ distribution with 2*k* degrees of freedom under the null hypotheses of the *k* tests. The *p*-value combined with the Fisher's combined probability test as denoted as $p_{c}^{p}$.

For each pathway *p*, *R*_*p*_ value was calculated to compare the relative significance of *G*_*a*_ and *G*_*i*_s dividing $p_{a}^{p}$ with $p_{c}^{p}$).

(5) $$B_{p}=log_{2}\left[\frac{log_{10}(p_{a}^{P})}{log_{10}(p_{c}^{P}))}\right]$$

#### 3.3.2. Cooperative Potential

The cooperative property of TFs was measured by comparing network centrality values between *G*_*all*_ and *G*_*i*_s (Equation 6). We used *betweenness centrality* of a node in a given sub-network that measures the proportion of the shortest paths present in the sub-network that pass through the corresponding node. Gene-level network centrality values were calculated on the *G*_*all*_ and the set of *G*_*i*_s, which are denoted as $c_{all}^{g}$ and $c_{i}^{g}$s. Then, the centrality value of the *G*_*all*_ ($c_{all}^{g}$) was divided by the square-rooted squared sum of $c_{i}^{g}$s. The cooperative potential of a pathway (*C*_*p*_) was calculated by summing up the cooperative potential of the overlap genes.

(6) $$C_{p}=\sum_{g\in P}\log_{2}\left[\frac{c_{all}^{g}}{\sqrt{\sum_{i=1}^{k}{\left(c_{i}^{g}\right)}^{2})}}\right]$$

### 3.4. Evaluation 3: Dynamics of GRNs Across Time

One of the advantages of our method is that the whole GRN is divided into small sub-networks. We suggest two approaches to choose sub-networks for detailed inspection.

- To emphasize on the dynamics of network over time, we chose sub-networks where regulatory relations vary significantly over time. For assessing the amount of variance across time, we measured the fraction of time-point exclusive nodes and edges to the size of a sub-network for each time point and then averaged across time. We applied this approach to the human dataset.
- To investigate how combinations of co-working TFs vary over time, we chose a Differentially Expressed Gene (DEG)-enriched TG module and inspected the DEG-enriched sub-networks connected to the TG module. We applied this approach to the *Arabidopsis thaliana* datasets. There were too many genes in the *Arabidopsis thaliana* network, thus we used only DEGs to reduce the number of genes.

## 4. Results

Given gene expression profiles, our method produces GRN that consists of multiple sub-networks where condition-specific interacting TFs regulate a set of TGs through intermediate genes. Utilizing the public GRN and signature genes as a guide, our method selects edges of the network with kernel CCA to model cooperative and combinatorial natures TFs and TGS. Another strength is that our method decomposes the whole GRN in to sub-networks to improve interpretability. When an organism is exposed to an environmental stimulus, it orchestrates multiple biological process as a response and what our method determines is the intermingled regulatory interactions. Therefore, decomposition of the whole GRN into sub-networks helps us to interpret the result better.

### 4.1. Comparative Analysis

We compared our method with the existing methods: ARACNe-AP (Lachmann et al., 2016) and GENIE3 (Irrthum et al., 2010). We compared how well each method can capture condition-specific network using GSE127530 dataset. Our method produced a set of TF–TG modules, i.e., a set of sub-networks, but existing methods produced a single network of large size. To compare the results, we combined a set of sub-networks from our method into a large single network. GENIE3 produced a set of million edges with importance score, and top 0.5% edges in terms of importance score were used for comparative analyses. To assess the performance of network inference, we retrieved 1131 HFM-related gene sets using a comprehensive biomedical entity search tool, BEST (Lee et al., 2016), as a condition-specific gene set (see section 3.2 for details). Both specificity and recall were used as metrics to compare the three methods for quantitative evaluation (Table 1). In node-level comparison, our method showed the best performance in terms of specificity and the second best in terms of recall in all time points. In edge-level comparison, our method showed the best performance in terms of both precision and recall in all time points. Additionally, in order to demonstrate that the non-linear technique for the construction of canonical components is necessary, we compared the performance of network inference by the regularized linear CCA with the performance of the regularized non-linear kernel CCA. In a majority of cases of performance comparisons, except recall of node comparison, utilizing kernel CCA exceeds in inferring the ground truth network.

**Table 1:** Comparison of our method to ARACNe-AP and GENIE3 in terms of specificity, precision, and recall with respect to the ground truth network from a literature search tool, BEST (Lee et al., 2016).

			**ARACNe-AP**	**GENIE3**	**Linear CCA**	**Kernel CCA**
+3 h	Node comparison	Specificity	0.841	0.270	0.692	0.961
		Recall	0.230	0.829	0.533	0.483
	Edge comparison	Precision	0	8.05 × 10^−6^	9.01 × 10^−3^	3.12 × 10^−2^
		Recall	0	9.04 × 10^−3^	0.413	0.591
+6 h	Node comparison	Specificity	0.869	0.277	0.741	0.957
		Recall	0.197	0.830	0.451	0.389
	Edge comparison	Precision	6.30 × 10^−6^	8.20 × 10^−6^	6.51 × 10^−4^	2.89 × 10^−2^
		Recall	8.84 × 10^−4^	9.04 × 10^−3^	0.188	0.340

### 4.2. Case Study 1: GRN in Response to HFM in Human

#### 4.2.1. Dynamics of GRN Over Time in Response to HFM

Dynamics of GRN over time in response to HFM was investigated. We executed our method on GSE127530 dataset obtaining a GRN for each time point (tp1 and tp2) with respect to tp0 as a baseline; a GRN with 7,021 nodes and 99,455 edges in tp1, and with 5,985 nodes and 61,646 edges in tp2. One challenge that arises in inspection of GRN is that regulatory relations are too complex to interpret in which multiple biological processes are intermingled together. One of the strengths of our method is that we can decompose the giant network into a feasible size of sub-network consisting of GRN projection from a TF module to a TG module. The resulting GRN from our method consisted of 31 TF modules and 76 TG modules in tp1 and 26 TF modules and 52 TG module in tp2 which means that 31 × 76 sub-networks and 26 × 52 sub-networks consists of a GRN of each time point.

**Figure 2:**
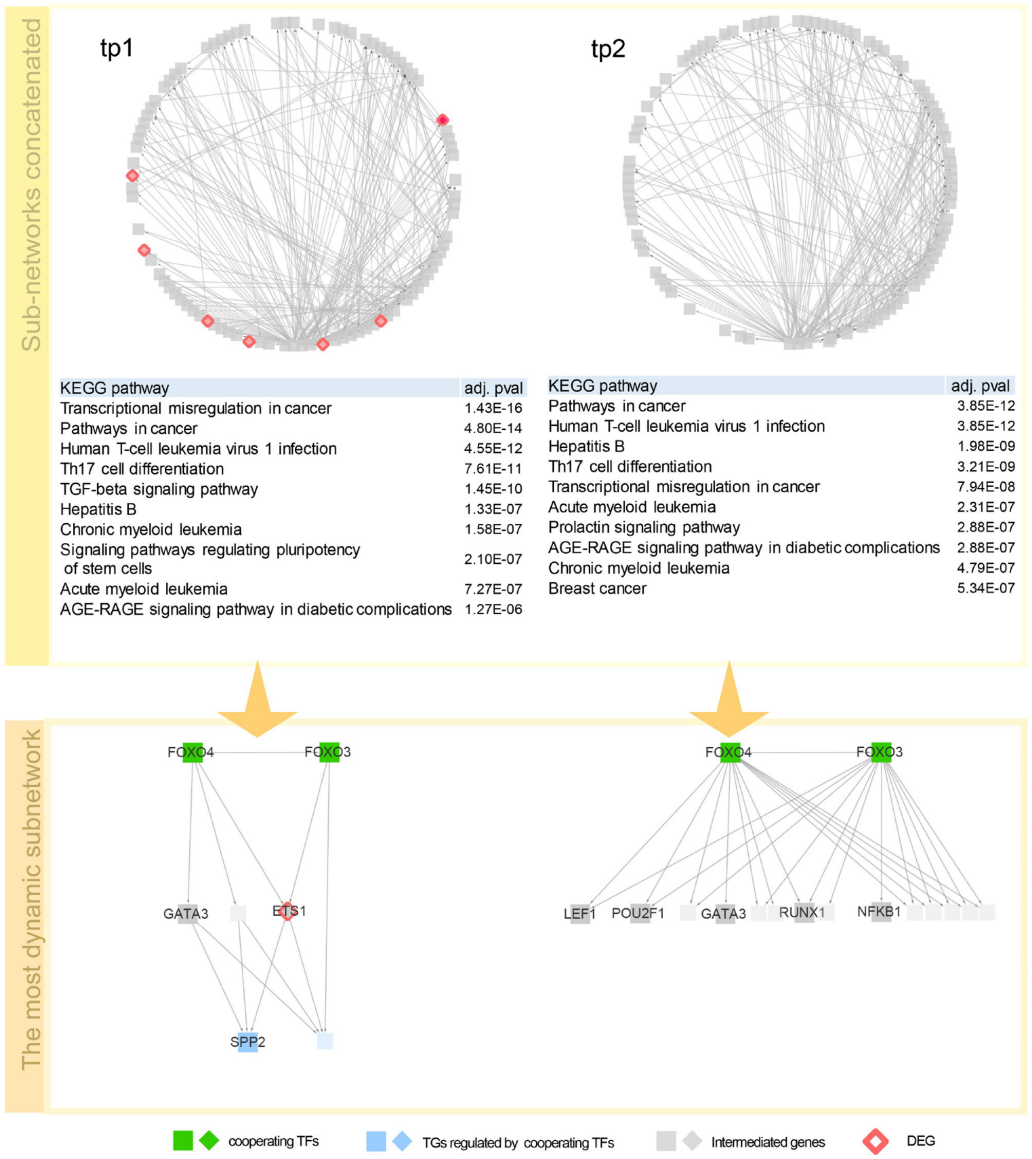
Network dynamics of a gene regulatory network (GRN) sub-network after high-fat meal (HFM) over time. Two circular diagrams in the upper panel show the change in gene–gene relationship, in particular, TF–TG regulation. Two tables in the middle summarize top 10 most enriched biological pathways with *p*-value corrected by false discovery rate (FDR) < 0.05. Two networks are dynamics of a TF–TG sub-network with the highest dynamics score. In the TF–TG sub-network, two transcription factors (TFs), FOXO3 and FOXO4, regulates different sets of target genes (TGs) over time. TFs were denoted with diamond-shaped nodes. Square nodes denotes TGs and circle nodes denote genes connected to TFs and TGs. Nodes colored pink denote genes that consist of GRN in each time point. Nodes colored red denote shared TFs among concatenated sub-networks and nodes colored green DEGs that are detected by DESeq with FDR < 0.05.

To investigate the regulatory mechanism over time, a network dynamics score of a TF–TG sub-network between two adjacent time points was measured. Basically, the score represents an average proportion of exclusiveness of genes at each time point. Detailed description of the score is given in section 3.4. With the score, we now can sort out TF–TG networks that show bigger change in network dynamics over time. By sorting TF–TG networks in terms of the score, we selected top 100 TF–TG networks. Each TF–TG network is a pair of a TF module and a TG module. Interestingly, many sub-networks shared common TF modules. Among 100 TF–TG networks, i.e., 100 pairs of a TF module and a TG module, 95 pairs of TF and TG modules share a TF module. With this observation, we can merge multiple TF–TG networks into single sub-networks. One sub-network that include 17 TG modules was used to investigate network dynamics over time after HFM—denoted as *G*_3*h*_ for tp1 and *G*_6*h*_ for tp2. We then compared how much biological pathways were enriched in these networks over time (Figure 2). As a result, immune system related pathways—Th17 differentiation, Th1 and Th2 cell differentiation, and inflammatory bowel disease (IBD)—were high ranked both in *G*_3*h*_ and *G*_6*h*_. Specifically, AGE-RAGE signaling pathway in diabetic complications was enriched in both time points. Advanced glycation end products (AGEs) and their receptor, RAGE, are known to deal with the accumulation of metabolite end product in diabetes (Ramasamy et al., 2011). The amount of soluble RAGE is also reported to play an important role in post-prandial response to HFM (Fuller et al., 2018).

Here are detailed discussions on dynamics of a TF–TG sub-network with the highest dynamics score (Figure 2). FOXO3 and FOXO4, which are the interacting TFs and are at the top hierarchy in TF cascading network, are isoforms of well-known nuclear TFs—FOXO family—that are involved in metabolic regulation (Barthel et al., 2005) and promoting inflammatory response in T cell (Kerdiles et al., 2010; Hedrick et al., 2012) implying the regulatory link between immune response and metabolic process. After 3 h after HFM, tumor necrosis factor α (TNF-α) and interleukin-6 (IL-6) are pro-inflammatory cytokines whose concentration reaches peak around 2–3 h after HFM (Herieka and Erridge, 2014). One of the TGs in the sub-network, S1P phosphatase 2 (SPP2) is known to play a pro-inflammatory role in induction of TNF-α and IL-6 (Mechtcheriakova et al., 2007). A differentially expressed gene, ETS1, encodes a TF involved in production of cytokine and chemokine in T helper cells (Russell and Garrett-Sinha, 2010; Garrett-Sinha, 2013) where one of the early responses of HFM is pro-inflammatory cytokine production. GATA3 is a family of GATA TF family that is an important regulator of T-cell development. According to Ibarra et al. (2020), FOXO1-ETS1 is reported as a potential cooperative TFs. FOXO1 and FOXO3 are the most dominant isotypes of Forkhead box family TF that coordinate common biological function—regulatory T cell development (Ohkura and Sakaguchi, 2010), implying that cooperative potential of FOXO regulation with ETS1 genes which is detected in our network. After 6 h after HFM, TF–TG relations that are regulating SPP2—one of the acute post-prandial responses—is diminished in the sub-network. However, other immune-responsive genes (i.e., POU2F1, RUNX1, NFKB1, and LEF1) are still enriched that are promoting other immune responses.

#### 4.2.2. Investigation of TF Cooperation in HFM

**Figure 3:**
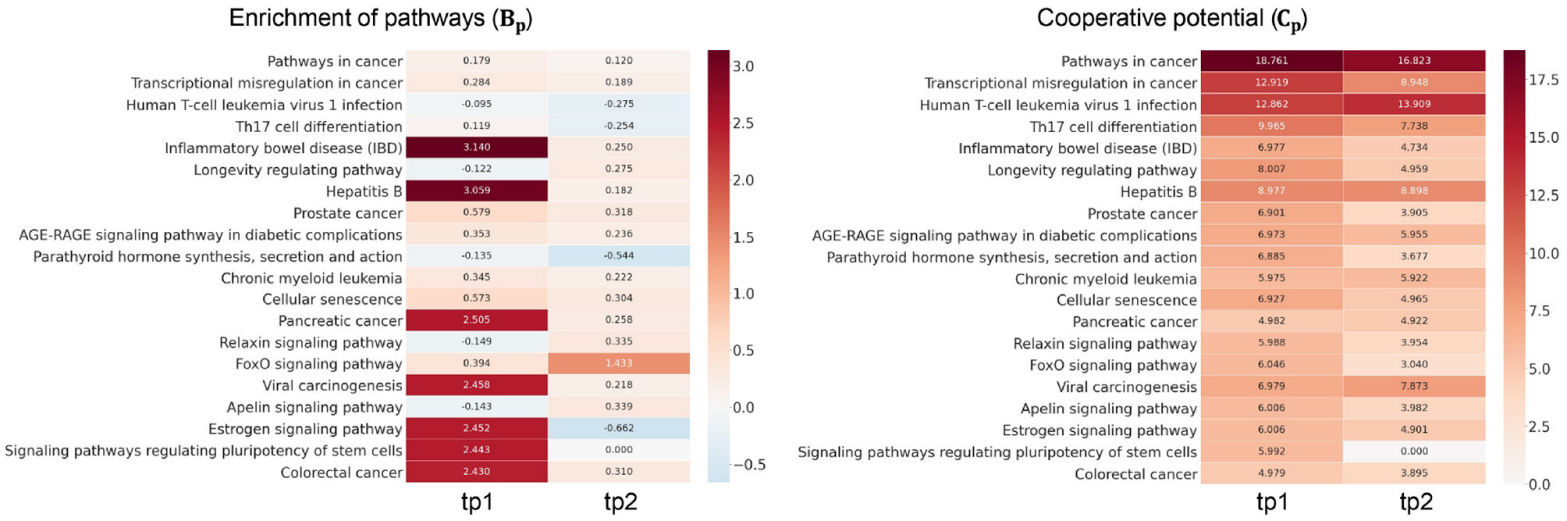
Examination of transcription factor (TF) cooperation. High score of *B*_*p*_ and *C*_*p*_ represents the amount of cooperativity of co-working TFs in the pathways. Heatmap in the left panel shows the cooperation in terms of pathway enrichment over time in high-fat meal (HFM). Pathway enrichment in the *G*_*all*_ was compared to the simulations given each TF (*G*_*i*_) and measured using Equation (5). Heatmap in the right panel shows the cooperative potential using enriched pathway genes. Betweenness centrality was compared between *G*_*all*_ and *G*_*i*_ using Equation (6).

We next investigated how much cooperation occurs in sub-networks (Figure 3). To demonstrate this, we analyzed the level of disruption if a single TF were considered—there are *n* simulations for *n* TFs in a given sub-network. To demonstrate this, we analyzed the level of disruption in pathways comparing sub-networks using multiple TFs (denoted as $G_{all}^{\prime }$) vs. simulated networks using individual TFs (denoted as $G_{i}^{\prime }$). The level of cooperation was measured at two perspectives: biological significance (*B*_*p*_) and cooperative potential (*C*_*p*_) between the $G_{all}^{\prime }$ and $G_{i}^{\prime }$.

The greater the *R*_*P*_ value is in a certain pathway *p*, the more genes exist in the *p* utilizing multiple TFs compared to the simulation with individual TFs. Heatmap in the left panel of Figure 3 depicts *B*_*p*_ value of the enriched pathways in $G_{all}^{\prime }$. Pathways including inflammatory bowel disease, hepatitits B, and estrogen signaling pathways showed greater TF cooperation at tp1. While at tp2, FOXO signaling pathway showed greater *B*_*p*_ at tp2 compared to that of the previous time point despite most of the pathways showed subtle enrichment changes against simulations. Such temporal changes indicate that there are regulatory dynamics in multiple pathways co-regulated by multiple TFs. *C*_*p*_ value in Equation (6) was developed here to investigate the degree of TF cooperation at $G_{all}^{\prime }$ in comparison to $G_{i}^{\prime }$ by summing up the individual contribution to cooperative potential of the genes in a sub-network. The greater betweenness centrality of a node is, the more shortest paths go through the node. As the whole network topology is more likely to be disrupted, the genes with high centrality are removed, and the node would play an important role in maintaining the given network topology.

*C*_*p*_ showed that tighter TF regulations were made by multiple TFs compared to the simulations throughout the enriched pathways. Compared to the subtle changes in *B*_*p*_ value, the ability of kernelCCA to construct a sensitive regulatory sub-network to temporal dynamics reflected greater *C*_*p*_ value across the pathways. Specifically, pathways related to cellular signaling were consistently co-regulated by two TFs (FOXO3 and FOXO4). This was also supported by a previous study that suggests greater co-regulation by multiple TFs stay invariant to perturbations as well as play a central role in controlling pivotal dynamics in response to external stimuli (Kim et al., 2012).

### 4.3. Case Study 2: GRN in Response to Heat and Cold Stress in *Arabidopsis thaliana*

#### 4.3.1. Dynamics of GRN Over Time in Response to Heat and Cold Stress

To investigate how combinations of co-working TFs vary over time, the sub-networks connected to the most DEG-enriched TG module are scope of our inspection. Since GRN of *Arabidopsis thaliana* is denser and more DEGs are detected than in human dataset, we, therefore, used DEG-centric approach that paths to DEGs from co-working TFs are inspected. All paths detected are listed in Appendix A.

It has been a long question how plants detect the lower and higher temperature and how they are sensing differences in the temperature. Usually, plants complete their whole life in one place where they germinated. Their growth undergoes diurnal rhythm and seasonal periodicity, which means the temperature condition is changing all the time. Plants can recognize the small change of temperature, such as 2–3 centigrade, called ambient temperature. The effects of these small changes are cumulative, having retention time to appear certain consequences even though the ranges of results vary depending on the stage of growth, other environmental conditions, and their genetic backgrounds. All of these processes occur in plants started from very minute changes at the molecular level. So, it has been an important task to undercover how plants recognize and trigger the serial and reversible and sometimes irreversible responses. Still, it is challenging to find out the group of genes in the thermal physiology of plants. We used two different Arabidopsis datasets, GSE5628 and GSE5621. GSE5628 represents heat stress that consists of heat-shocked samples up to 3 h at 38 centigrade and recovered samples after heat-shock treatment prolongs to 21 h at 25 centigrade. When outranged thermal changes have occurred, all responses of plants go for stabilizing homeostasis.

**Figure 4:**
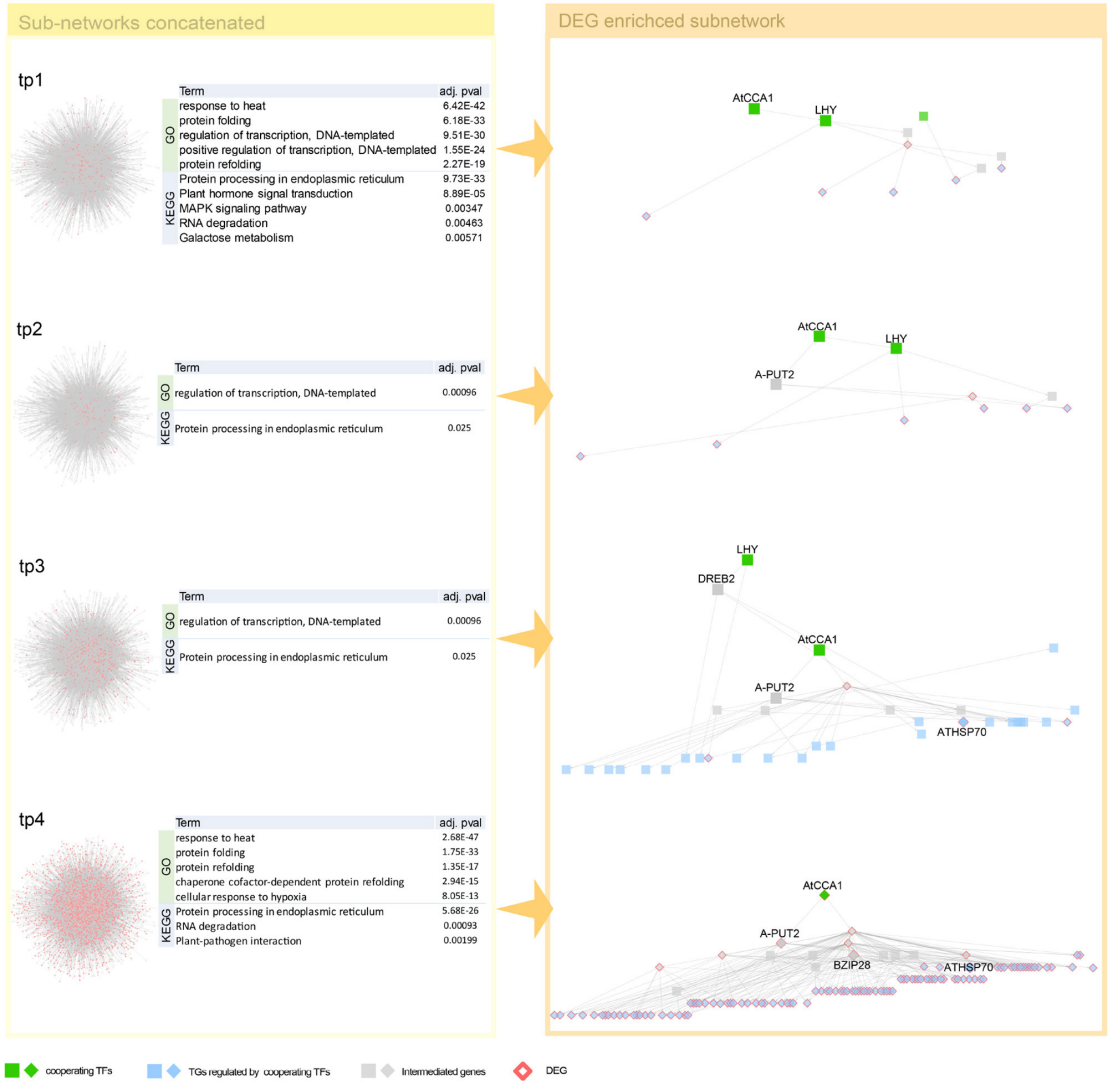
Network dynamics of a gene regulatory network (GRN) sub-network after heat stress over time. Network in the left panel shows the change in gene-gene relationship, in particular, TF–TG regulation. DEGs are denoted as pink. Four tables in the middle summarize top five most enriched biological pathways with *p*-value corrected by false discovery rate (FDR) < 0.05. Heat stress related Gene Ontology (GO) terms are enriched in GO enrichment tests with DEGs. The networks in the right panel are dynamics of a TF–TG sub-network that are DEG enriched. Transcription factors (TFs) were denoted with green nodes. Blue nodes denote target genes (TGs) and gray nodes denote genes connected to TFs and TGs. Square nodes denote DEGs that are detected by Limma with FDR < 0.05.

Interestingly, we detected both genes of circadian clock associated (CCA1) and late elongated hypocotyl (LHY), a short period after high-temperature treatment. These two genes, detected as a co-working TFs in our proposed method (Figure 4), involve in common biological pathway—a central role in the phytochrome-medicated circadian clock (Alabadi et al., 2002; Dong et al., 2011). After that, we observed in one of our early-stage tp1 and tp2 of heat-path various TCP genes, PIF5 (PUT2) and CAT genes, those involved in thermosensory (Michael et al., 2003; Zhou et al., 2019; Balcerowicz, 2020). A path of phytochrome-mediated thermo-response appears tp3 stage. Mainly PIF4 and many of its downstream genes include directly related genes, such as TCPs and BZIP28 and indirectly related genes that mediate heat shock responses (Che et al., 2010).

**Figure 5:**
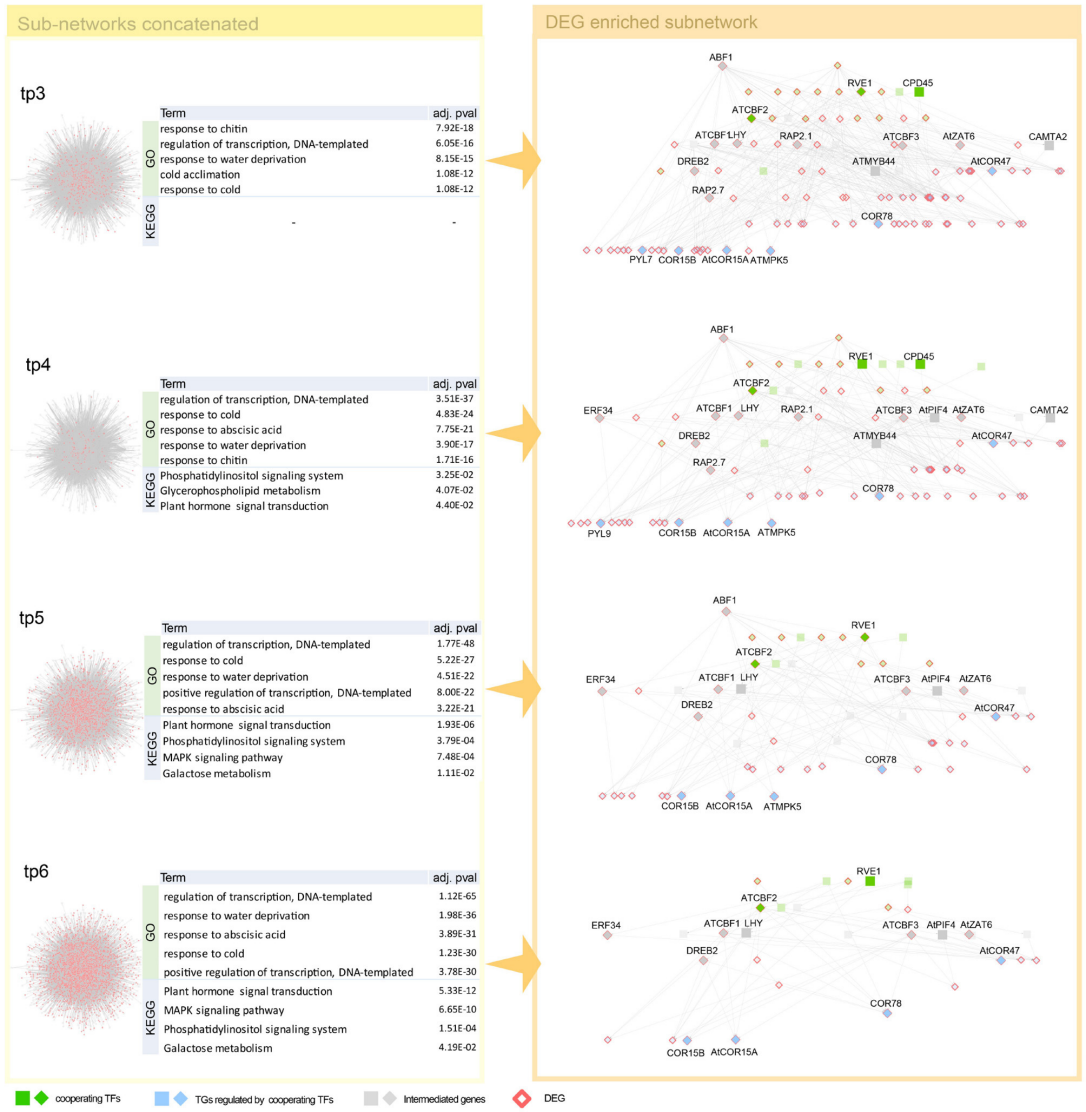
Network dynamics of a gene regulatory network (GRN) sub-network after cold stress over time. Networks in the left panel shows the change in gene–gene relationship, in particular, TF–TG regulation. DEGs are denoted as pink. Four tables in the middle summarize top five most enriched biological pathways with *p*-value corrected by false discovery rate (FDR) < 0.05. Cold stress related Gene Ontology (GO) terms are enriched in GO enrichment tests with DEGs. The networks in the right panel are dynamics of a TF–TG sub-network that are DEG enriched. TFs were denoted with green nodes. Blue nodes denote TGs and gray nodes denote genes connected to transcription factors (TFs) and target genes (TGs). Square nodes denote DEGs that are detected by limma with FDR < 0.05. We showed GRN from tp3 to tp6, since GRN constructed in tp1 and tp2 is too small because the number of DEGs are too small in tp1 and tp2—41 and 23, respectively.

Unlike a higher temperature treatment for several hours that increases physiological reactions and results in less severe consequences, lower temperature treatment over hours is life threatening. This characteristic difference of temperature treatment is why we found a relatively broad range of gene regulatory paths from cold treatment. We found well-defined cold response genes, such as CBF, DREB, COR, ERF, ZAT, RVE, and ABF1 (Vogel et al., 2005; Lee and Thomashow, 2012; Meissner et al., 2013; Wang et al., 2017; Dubois et al., 2018) and many cold stress-related genes from the early stage of cold treatment (Figure 5, Appendix A). Co-working TFs, such as RVE1, CPD45, and ATCBF2, detected in our GRN are involved in common cold related pathways implying cooperative functions of the TFs (Eremina et al., 2016; Chen et al., 2020). We found CCA1, LHY, and PIF4 gene from DEGs of cold temperature treated samples (Figure 5). It might have resulted from the thermosensory networkś change even though the treatmentś degree was far beyond the ambient temperature to the lower direction. It might be noteworthy that we observed the genes of developmental processes like RVE and cold acclimation related COR and CBF. There are several reports on CBF gene regulation. We found most of CBF promoter binding TFs, such as PIFs, CCA1, and LHY (Dong et al., 2011; Jiang et al., 2017). Several genes reported as intermediate genes—connecting the co-working TFs and the DEGs regulated by the TFs—are involved in common cold responsive pathways, implying that cooperative action of regulating downstream DEGs (Appendix A).

### 4.4. Discussion and Conclusion

In this paper, we proposed a kernel CCA based condition-specific GRN inference method that models combinatorial and cooperative nature of TF–TG relations. The traditional approach is to start with the whole network and test validity of edges, which lead to a condition-specific network based on gene expression data. One major issue with this approach is to deal with a single large network as a whole, which is challenging. However, we know that each TF regulates a relatively small number of genes, typically several hundred genes. So, it is possible to limit the scope of TGs that are regulated by a single TF. In fact, experimental techniques, such as TF ChIP-seq provide condition-specific comprehensive snapshot of genes that are targeted by a TF. Although these experimental data provides condition-specific targets of a TF, there are two major issues for utilizing such TF ChIP-seq data. First, a TF ChIP-seq experiment provides TGs of the TF only. Since TF may target different genes under different conditions, reconstruction of condition-specific networks requires TF ChIP-seq experiments for “all” relevant TFs, which is infeasible due to the time and budget constraints. Second, even if we can perform such expensive experiments, we need to combine many TF-networks into large networks. One major issue for this task is to identify co-operating TFs in a specific condition, but this is largely unknown.

#### 4.4.1. Advantages and Limitations

The novelty of our approach is to address the two issues in a single computational framework. First, we used clustering approach to reduce the search space by generating a set of TF clusters and a set of TG clusters. This approach allows us handle much smaller networks. Specifically, a TF set vs. a TG set is considered one at a time. Second, use of kernel CCA allows us to investigate on the complex relationship of multiple TFs vs. multiple TGs. In the final step of our computational framework, all TG sets that are related to a single TF set are merged, which generates condition-specific sub-networks. By performing analysis on transcriptome of human high-fat data and of arabidopsis cold and heat data at each time point, temporal dynamics of TF–TG networks was constructed by explaining condition-specific biological mechanisms successfully.

Although our method was successful in constructing dynamics of condition-specific TF–TG networks over time in both data sets, there are several issues remaining as further study. In the current framework, clustering of TF and TG modules need more rigorous definitions. The size of TF and TG modules vary greatly—some clusters consist of few genes while others consists of hundred genes. Merging TF–TG sub-networks in the final step of our method also need more rigorous guideline. We suggested two approaches in selecting sub-networks that show condition-specific response, which is meaningful, but there is still room for improvement to consider the non-responsive gene regulatory interactions that are required for fundamental cellular functions.

In terms of biological perspectives, there are also several issues that requires further study. First, our method does not discriminate stimulative or repressive gene regulation. Another issue is with kernel CCA. Kernel CCA can detect multiple-to-multiple relations of TFs and TGs, it does not discriminate whether correlations are positive or negative. In addition, our method assume that TF is a major regulator. However, there are other regulatory mechanisms, such as mutations, copy number variations, and epigenetic mechanisms, that can affect transcription level of genes. This requires a comprehensive model, e.g., ensemble of deep learning (Kang et al., 2020). Combining network analysis techniques and deep learning technologies is a major current research topic.

## Data Availability Statement

Publicly available datasets were analyzed in this study. RNA-seq data of human blood transcriptome analyzed in this study can be found in the GEO (https://www.ncbi.nlm.nih.gov/geo/query/acc.cgi?acc=GSE127530) and microarray data of Arabidopsis thaliana can be found in the GEO (https://www.ncbi.nlm.nih.gov/geo/query/acc.cgi?acc=GSE5621; https://www.ncbi.nlm.nih.gov/geo/query/acc.cgi?acc=GSE5628). Code for GRN construction with kernel CCA can be found at: https://github.com/DabinJeong/GRN_construction_with_kernelCCA (Mölder et al., 2021).

## Author Contributions

SK designed and directed the whole project. DJ designed and implemented the GRN construction algorithm. SLi, MO, and SLe involved in the discussion for building thesis. SLi designed the demonstration strategy and visualized the results. CC conducted the comparison analysis. WJ, SLi, DJ, SLe, MO, and CC biologically interpreted the analysis results. SK, DJ, and SLi wrote and revised the paper. All authors contributed to the article and approved the submitted version.

## Conflict of Interest

The authors declare that the research was conducted in the absence of any commercial or financial relationships that could be construed as a potential conflict of interest.
